# The number of domains in the ribosomal protein S1 as a hallmark of the phylogenetic grouping of bacteria

**DOI:** 10.1371/journal.pone.0221370

**Published:** 2019-08-22

**Authors:** Andrey V. Machulin, Evgenia I. Deryusheva, Olga M. Selivanova, Oxana V. Galzitskaya

**Affiliations:** 1 Skryabin Institute of Biochemistry and Physiology of Microorganisms, Federal Research Center “Pushchino Scientific Center for Biological Research of the Russian Academy of Sciences”, Pushchino, Moscow Region, Russia; 2 Institute for Biological Instrumentation, Federal Research Center “Pushchino Scientific Center for Biological Research of the Russian Academy of Sciences”, Pushchino, Moscow Region, Russia; 3 Institute of Protein Research, Russian Academy of Sciences, Pushchino, Moscow Region, Russia; 4 Institute of Theoretical and Experimental Biophysics, Russian Academy of Sciences, Pushchino, Moscow Region, Russia; University of Queensland, AUSTRALIA

## Abstract

The family of ribosomal proteins S1 contains about 20% of all bacterial proteins including the S1 domain. An important feature of this family is multiple copies of structural domains in bacteria, the number of which changes in a strictly limited range from one to six. In this study, the automated exhaustive analysis of 1453 sequences of S1 allowed us to demonstrate that the number of domains in S1 is a distinctive characteristic for phylogenetic bacterial grouping in main phyla. 1453 sequences of S1 were identified in 25 out of 30 different phyla according to the List of Prokaryotic Names with Standing in Nomenclature. About 62% of all records are identified as six-domain S1 proteins, which belong to phylum Proteobacteria. Four-domain S1 are identified mainly in proteins from phylum Firmicutes and Actinobacteria. Records belonging to these phyla are 33% of all records. The least represented two-domain S1 are about 0.6% of all records. The third and fourth domains for the most representative four- and six-domain S1 have the highest percentage of identity with the S1 domain from polynucleotide phosphorylase and S1 domains from one-domain S1. In addition, for these groups, the central part of S1 (the third domain) is more conserved than the terminal domains.

## Introduction

A comprehensive investigation of the distribution of ribosomal proteins and finding of the specific signatures of ribosomal evolution between and within the ribosomal protein domains is an actual task, which provides new insights into the emergence and evolution of the protein component of ribosomes [1–4].

**Fig 1:**
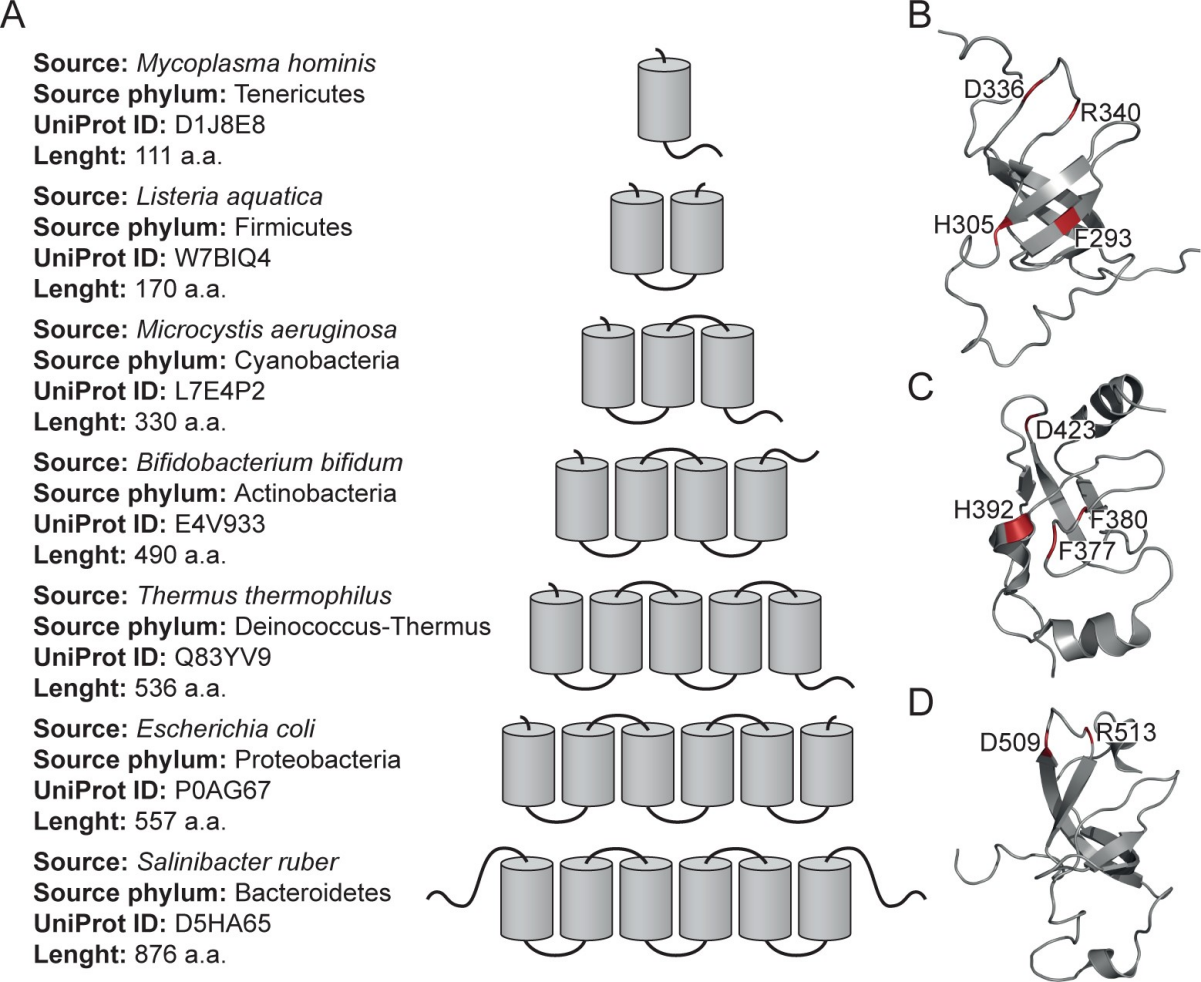
S1 domains in bacteria. (A) Number of structural S1 domains in different bacteria (according to the SMART database). (B), (C), (D) NMR structures of the fourth (2KHI), fifth (5XQ5) and sixth (2KHJ) S1 domains from *E*.*coli*. Conserved residues are located on the surface of the domains are given.

As demonstrated in our recent paper [5], the family of polyfunctional ribosomal proteins S1 contains about 20% of all bacterial proteins, including the S1 domain. This domain is one of the structural versions of the OB-fold (oligonucleotide/oligosaccharide-binding fold), which is considered to be one of the “most ancient” protein folds tolerant to mutations (designable) and able to accommodate to the binding of a large number of ligands [6,7]. Proteins of this family interact with mRNAs, are involved in initiation and translation of mRNAs *in vivo* and interact with the mRNA-like part of the tmRNA molecule [8,9]. Like some other ribosomal proteins, ribosomal protein S1 is an autogenic repressor of its own synthesis [10]. In addition, S1 can perform functions outside of the ribosome. For example, the S1 domain as a part of one of the four subunits of phage Qβ replicase increases its activity upon interaction with the ribonuclease regB of bacteriophage T4 [11,12]. Initially, in the ribosomal protein S1 from *Escherichia coli*, four unique repeats were found [13]. This repeat was named the S1 RNA-binding motif or the S1 domain. Later, using the protein sequence alignment, six homologous repeats were identified in ribosomal protein S1 (*E*. *coli*) [14]. The following studies demonstrated that the number of structural S1 domains in bacteria changes strictly within a limited range from one to six (Fig 1A).

At present, the structure of S1 from *E*. *coli* was obtained only with a very low resolution of 11.5 Å using cryo-electron microscopy [15]. Detailed 3D structure is not determined due to the high flexibility of S1 [16]. In the Protein Data Bank, there are only 3D structures of separate domains of S1 from *E*. *coli* obtained by NMR [17]. The RNA-binding domain of S1 is a β-barrel with an additional α-helix between the third and fourth β-strands (Fig 1B–1D). Consequently, the understanding of the connection between S1 repeats and their evolution, functions, and structures is a significant task.

Domain S1 is one of the “most ancient” protein domains, and therefore its presence in different combinations is a direct result of the evolution of microorganisms [6,7]. For example, the number and pairwise alignment of S1 domains in the family of ribosomal proteins S1 were used [17] to probe the relationship between S1 proteins of Gram-negative and Gram-positive bacteria. The study was performed considering 26 bacteria, which are typical representatives of its phylum. The other authors [18] used the rpsA gene, which codes the ribosomal protein S1, as a biomarker for identification of differences between 8 types of mycobacteria, which were not disclosed by the analysis of the 16S RNA.

In this study, the automated advanced exhaustive search allowed us to obtain a large dataset including 1453 sequences of S1 proteins. We performed the bioinformatics analysis of 1453 sequences of S1 and demonstrated that the number of structural domains in the family of ribosomal proteins S1 is a distinctive characteristic for phylogenetic bacterial grouping in main phyla. The alignments of S1 sequences with the S1 domain from polynucleotide phosphorylase (PNPase) and other proteins S1 containing one structural domain allowed us to find the sequential number of the domain with the highest value of identity and the largest number of representatives. In addition, we showed that the central part for some groups of S1 (proteins with five and six domains) is more conserved than the terminal parts.

## Results

### Databases of structural domains

The automated advanced exhaustive analysis allowed us to choose 1453 records corresponding to search parameters. As noted above (see Materials and Methods), for each analyzed record in the studied list, the numbers of S1 domains were collected from four databases of structural protein domains: SMART, Pfam, PROSITE, and SUPFAM. Data on all identified domains and their numbers from these databases are reflected in each record in the UniProt database. Each database has definite algorithms for providing information about protein structures, their folds, and domain organization. Inasmuch as in some cases, these algorithms are different and the databases with the same algorithm have usually additional conditions or restrictions, the output data may differ for the same object [19]. Therefore, we analyzed data on representation (the number of different records) for the family of ribosomal proteins S1 in the considered databases of protein domains (S1 Table). The data for the family of ribosomal proteins S1 for the analyzed phyla of bacteria are represented equally in the four databases of protein domains, showing that the analyzed collection is complete and corresponds to the aim of the study.

### Phylogenetic bacterial grouping

**Fig 2:**
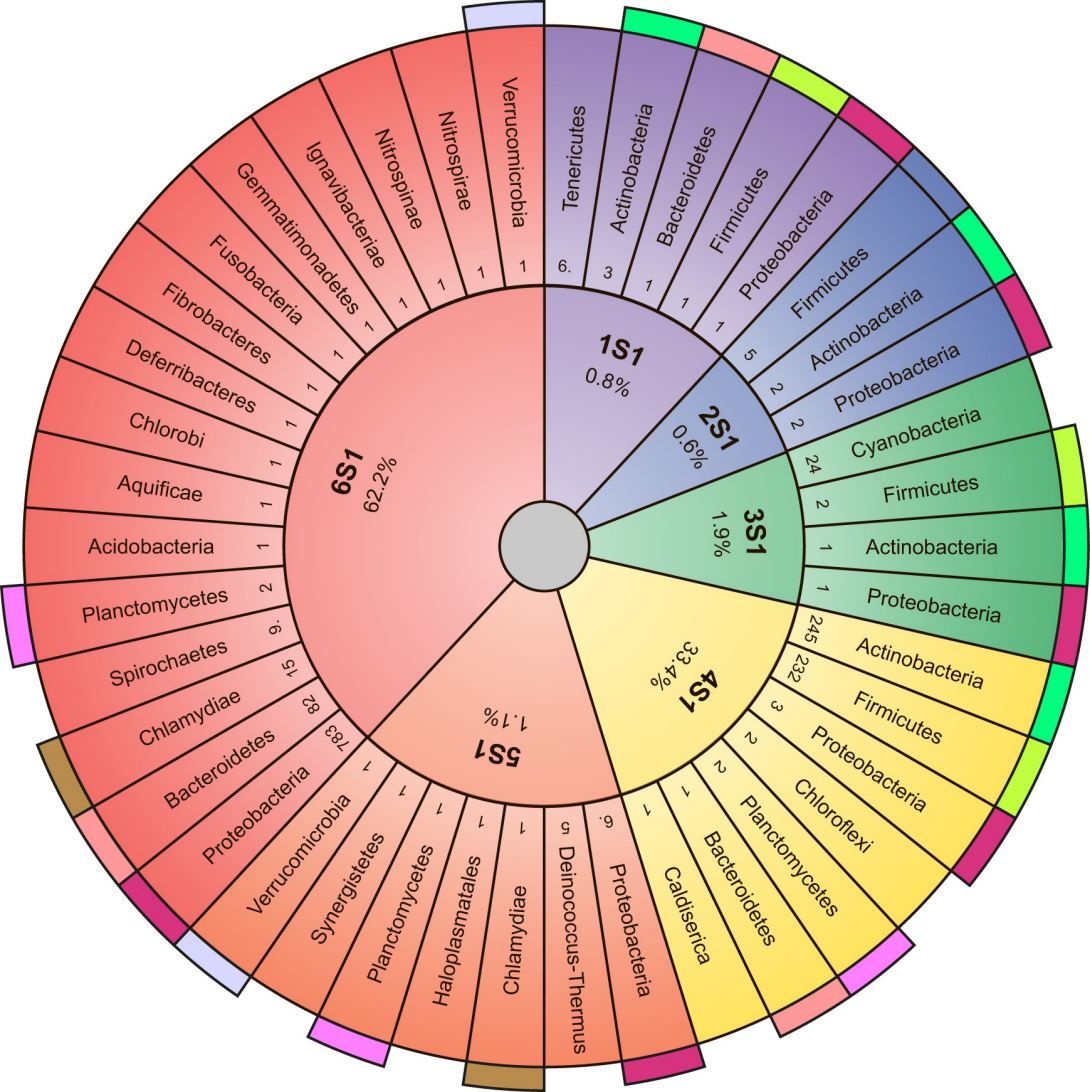
Sunburst chart reflecting division of studied bacteria into phyla. Names of phyla correspond to the Taxonomy Browser. Colored regions show bacteria containing the same number of domains of ribosomal protein S1 in the different phyla (from one to six). Colored outer segments represent the same phyla. Numbers, which are located near the phylum name, correspond to the numbers of representatives of records for each phylum.

The 1453 sequences of S1 satisfying our selection criteria were referred to the final dataset for bioinformatics analysis. At present, all Bacteria are divided into about 30 main phyla (List of Prokaryotic Names with Standing in Nomenclature (LPSN), http://www.bacterio.net/). Some phyla include only several types of bacteria (for example, Thermomicrobia, Chrysiogenetes, Fibrobacteres, Deferribacters), while about 90–95% of all known bacteria are included in such phyla as Proteobacteria, Bacteroidetes, Cyanobacteria, Actinobacteria and Firmicutes. The 1453 S1 sequences were identified in 25 different phyla (except candidate phyla). All studied phyla of bacteria and the number of S1 domains found in them are shown in the sunburst chart (Fig 2).

**Fig 3:**
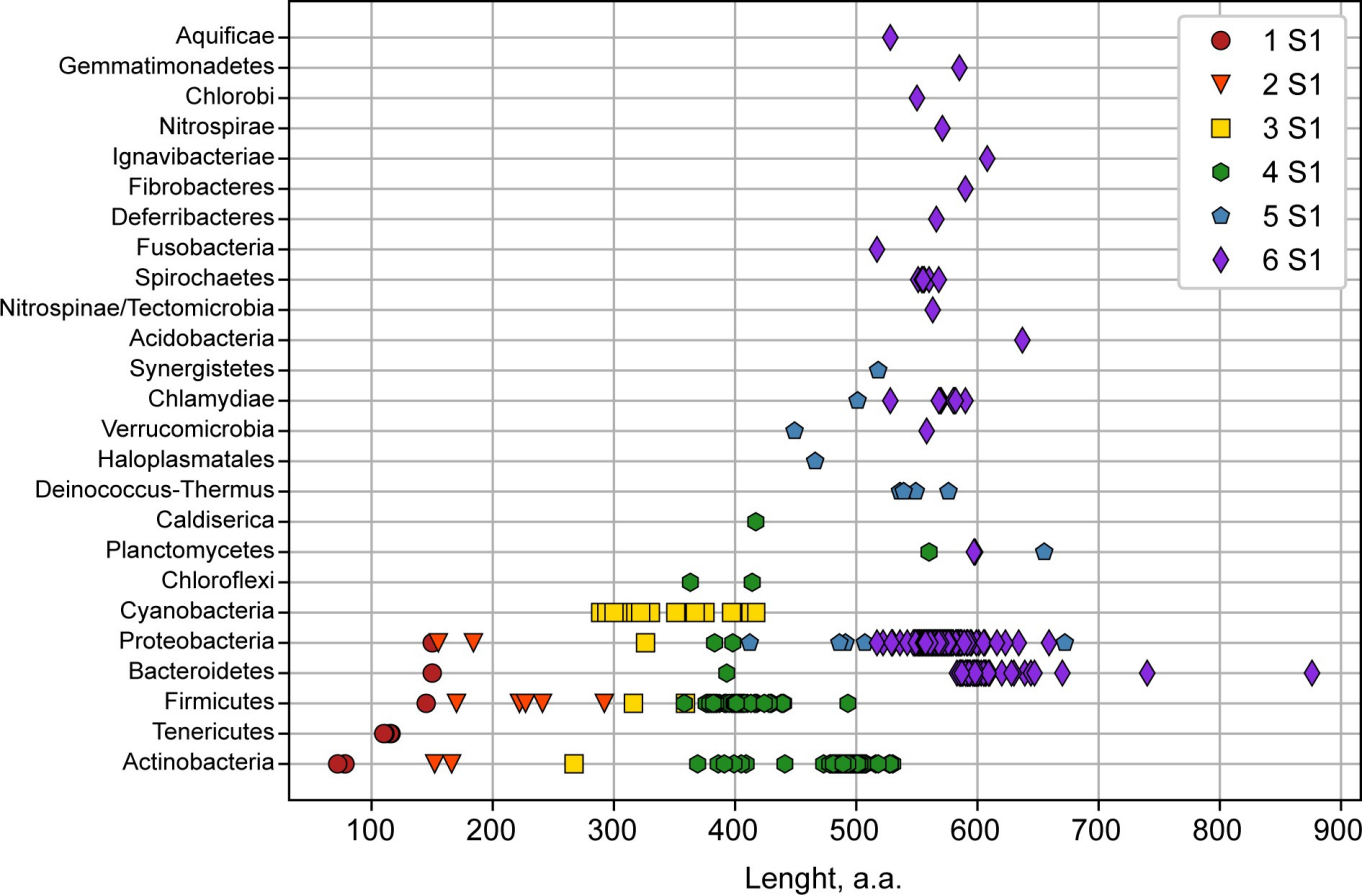
Phylogenetic bacterial grouping by the numbers of structural domains in the family of ribosomal proteins S1. Colored symbols show bacteria containing the same number of S1 domains in the ribosomal protein S1 (from one to six).

Only 0.8% from all investigated ribosomal proteins S1 contain one S1 domain. The most represented in this group is the phylum Tenericutes. It should be noted that mycoplasma is the simplest independent reproducing living organism. The total amount of its genetic information is four times less than that of *E*. *coli* [20]. The shortest full length S1 is found in members of the Mycoplasmatacea family (*Mycoplasma auris*– 110 amino acid residues). One S1 domain is also found in very few bacteria of phylum Actinobacteria. One S1 domain is also identified in some bacteria of phyla Firmicutes, Proteobacteria, and Bacteroidetes. Interestingly, in all studied phyla only several bacteria (0.6%) containing two S1 domains were found (some bacteria from phyla Actinobacteria, Firmicutes, and Proteobacteria). In all cases, Cyanobacteria with an average length of ribosomal protein S1 of about 350 amino acid residues have three S1 domains; also, some representatives of phyla Firmicutes, Actinobacteria, and Proteobacteria have three-domain S1 proteins. Generally, three-domain S1 proteins are identified in 1.9% cases. Records with four S1 domains were identified in 33% cases from all investigated ribosomal proteins S1. Almost all analyzed bacteria (with the protein length of 390 amino acid residues) in this group relate to phyla Actinobacteria (50% from all four-domain S1 proteins) and Firmicutes (47% from all four-domain S1 proteins). In bacteria of the monotypic (consisting of one Deinococci class) phylum Deinococcus-Thermus, the length of protein S1 is on average about 530 amino acid residues, and these bacteria have always five S1 domains (31% from all five-domain S1 proteins). Five S1 domains are also found in bacteria of phyla Thermotogae, Synergistetes, Haloplasmatales. Generally, five-domain S1 proteins compose 1.1% from all investigated ribosomal proteins S1. About 62% of the records are identified as proteins containing six S1 domains. Generally, these proteins belong to phylum Proteobacteria. Ribosomal proteins S1 from bacteria of the phylum Chlorobi (green sulfur bacteria) also have six S1 domains. Gram-negative bacteria containing six S1 domains include Spirochaetes, Bacteroidetes, Chlamidia, and Proteobacteria (α, β, γ, δ, ε). In these bacteria, the length of the ribosomal protein S1 averages about 570 amino acid residues. Gram-positive bacteria contain different numbers of the S1 domain depending on the phylum. Phylogenetic bacterial grouping according to the number of structural domains and length considering 1453 S1 sequences is shown in Fig 3. As can be seen, the number of S1 structural domains in the family of ribosomal proteins S1 varies in a strictly limited range from one to six.

### Comparison of the number of structural S1 domains in the family of ribosomal proteins S1 identified by different methods

The automated advanced exhaustive analysis of 1453 S1 sequences allowed us to demonstrate that the number of structural domains in S1 is a distinctive feature (hallmark) for the phylogenetic grouping of bacteria in the main phyla. Several attempts have been made to classify ribosomal protein S1 according to a different number of sequences. We are the first who has performed an exhaustive analysis of S1. As noted above, S1 is identified in 25 different phyla. 13 phyla were studied by Salah et al. [17] They used the number and pairwise alignment of S1 domains in the family of ribosomal proteins S1 to study the relationship between Gram-positive and Gram-negative bacteria. 12 phyla were identified considering 273 S1 sequences[19]. Considering 1453 S1 sequences we found that four-domain proteins predominate in the phylum Firmicutes. According to our data, a large number of bacteria of the phylum Actinobacteria contain four S1 domains and a smaller number contains five S1 domains. The authors of the cited paper [17] refer all Actinobacteria to five-domain proteins with one domain at the C-terminus, which is not identified as the S1 domain. Moreover, itcan be seen that the phylum Proteobacteria contains all possible versions of the number of S1 domains with predominant six domain proteins (Fig 3). It should be noted that each domain in S1 play different roles. So, for a well-studied bacterial 30S ribosomal protein S1 from *E*.*coli*, the biochemical experimental study of various fragments allowed to establish the functions of individual protein domains and parts. It was shown that the removal of one S1 domain from the C-terminus or two S1 domains from the N-terminus of a protein decreases only the effectiveness of the protein functions, but not its functional capabilities [21,22]. Wherein, for example, the bacterial 30S ribosomal protein S1, which has only one domain of parasitic bacteria of Mollicutes, effectively performs the main function of RNA-binding [23].

### Grouping of bacterial phyla in superphyla and the number of S1 domains

At present the evolutionary development and affinity of most bacterial phyla has remained unclear, but some phyla were grouped into superphyla using a number of features. For example, phylum Bacteroidetes is sometimes grouped with phyla Chlorobi, Fibrobacteres, Gemmatimonadates, and Ignavibacteriae in the FCB group [24]. Our data from list (http://bioinfo.protres.ru/other/Amount_and_borders_S1_domain.xlsx) demonstrate that the ribosomal protein S1 in this group always contains six S1 domains (Fig 3).

It should be noted that these phyla on phylogenetic trees are often on the same level, that is, they evolved evolutionarily in parallel. Analysis of 16S rRNA and characteristic conserved indels in some proteins is used to group phyla Planctomycetes, Verrucomicrobia, Chlamydiae in the PVC clan [25]. As shown by our data (Fig 3), bacteria of the phyla Chlamydiae and Verrucomicrobia mainly contain six S1 domains, while Planctomycetes can have four, five, and six S1 domains. According to some published data, the genome of organisms of the phylum Planctomycetes compared with other phyla of superphylum PVC is the largest and most susceptible to evolutionary changes [26].

### Family of 30S ribosomal protein S1 and RNA-binding S1 domain of polynucleotide phosphorylase

It is known that the polynucleotide phosphorylase (PNPase) from *E*. *coli* contains at its C-end one S1 domain with high identity to the initially isolated four S1 repeats. The 3D structure of the S1 RNA binding domain from *E*. *coli* PNPase obtained by NMR spectroscopy is a β-barrel with an additional α helix between the third and fourth β strands [6]. This OB-fold (oligonucleotide binding fold) is generally considered as the main structural element of the ribosomal protein S1 family [27].

**Fig 4:**
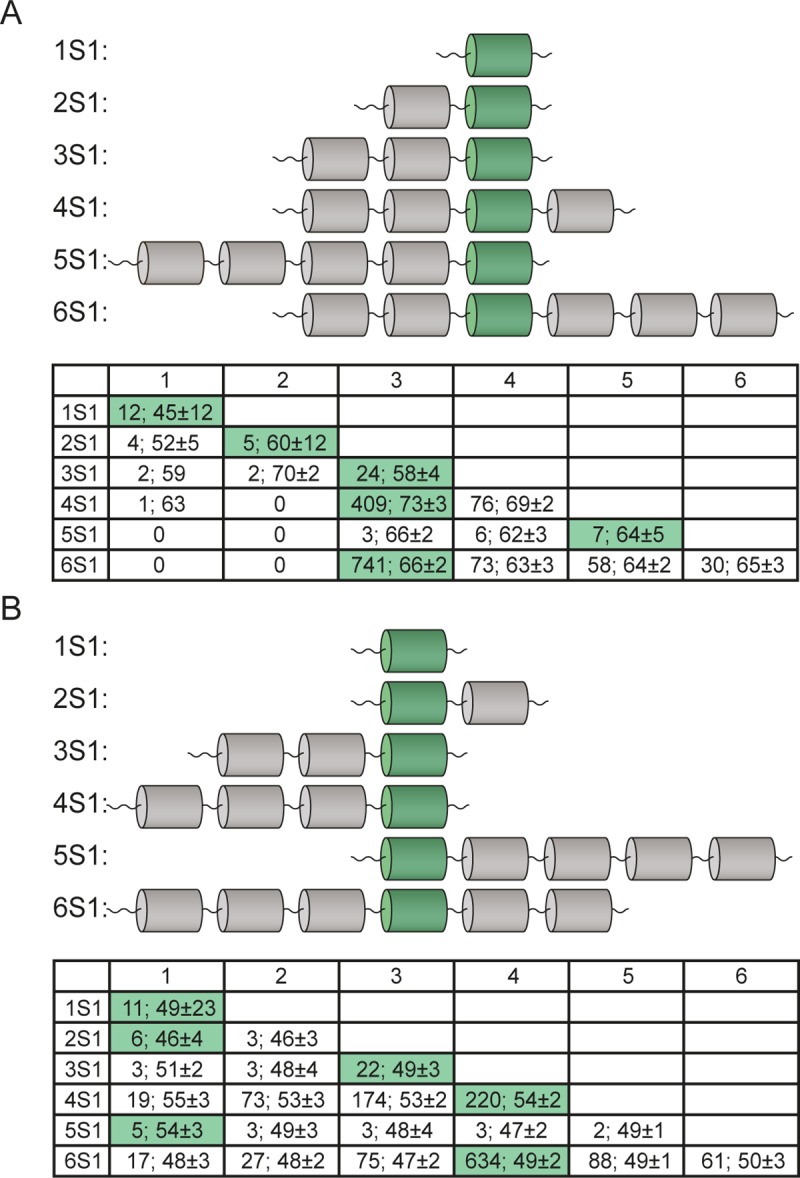
S1 containing different numbers of domains. The conserved domains with the highest identity to (A) S1 domain from PNPase and (B) S1 domain from *M*. *auris* are shown in green color (UnitProt ID: N9VCN6).

Alignments of 1453 S1 sequences and the S1 domain of PNPase allowed us to calculate the average identity for each S1 domains and find the sequence number (No.) of the domain with the high value of identity with the RNA-binding S1 domain of PNPase [2] with the most representatives. This domain migrates along the chain (Fig 4A). This is the last domain in two-, three-, and five-domain proteins. For most representatives of four- and six-domain proteins the highest value of identity with PNPase and the amount of representatives were found for the third domain (for four-domain proteins: 409 records with 73% identity and for six-domain proteins: 741 records with 66% identity).

In addition, for the four- and six-domain S1 proteins, the fourth domains are also have a high value of identity with PNPase and a large number of representatives. It should be noted that the residues Phe19, Phe22, His34, Asp64, and Arg68 [28] (in some cases replaced by the corresponding conserved residues) are located in this particular conserved domain, which once again confirms the uniqueness of this repeat and should be considered as the strongest RNA binding site [28].

Alignments of the S1 sequences and the S1 domain protein from *M*. *auris* (the shortest full length S1 protein with 110 amino acid residues) allow us to find the domain with the highest value of identity and the largest number of representatives located in the third domain (Fig 4B). For four- and six-domain proteins, the highest value of identity with the S1 domain protein from *M*. *auris* and the number of representatives were found for the fourth domain (for four-domain proteins: 220 records with 54% identity and for six-containing domains: 634 records with 49% identity). The same correlation (the third or fourth domain with the highest value of identity and number of representatives) is found when the S1 protein sequences are aligned with other one-domain S1 proteins (UniProt IDs: B3PLZ6, I5D611, N9UB66, D1J8E8, Q6KH89; http://bioinfo.protres.ru/other/homology_with_PNPase_and_one-domain%20S1.xlsx).

Thus, the most conserved domains with the S1 domain from PNPase (*E*.*coli*) and S1 domains from one-domain S1 proteins (mainly Tenericutes, Mollicutes) are the third and fourth domains for the most representative four- and six-domain S1 proteins.

### Search for the conserved domain within the family of 30S ribosomal protein S1

**Fig 5:**
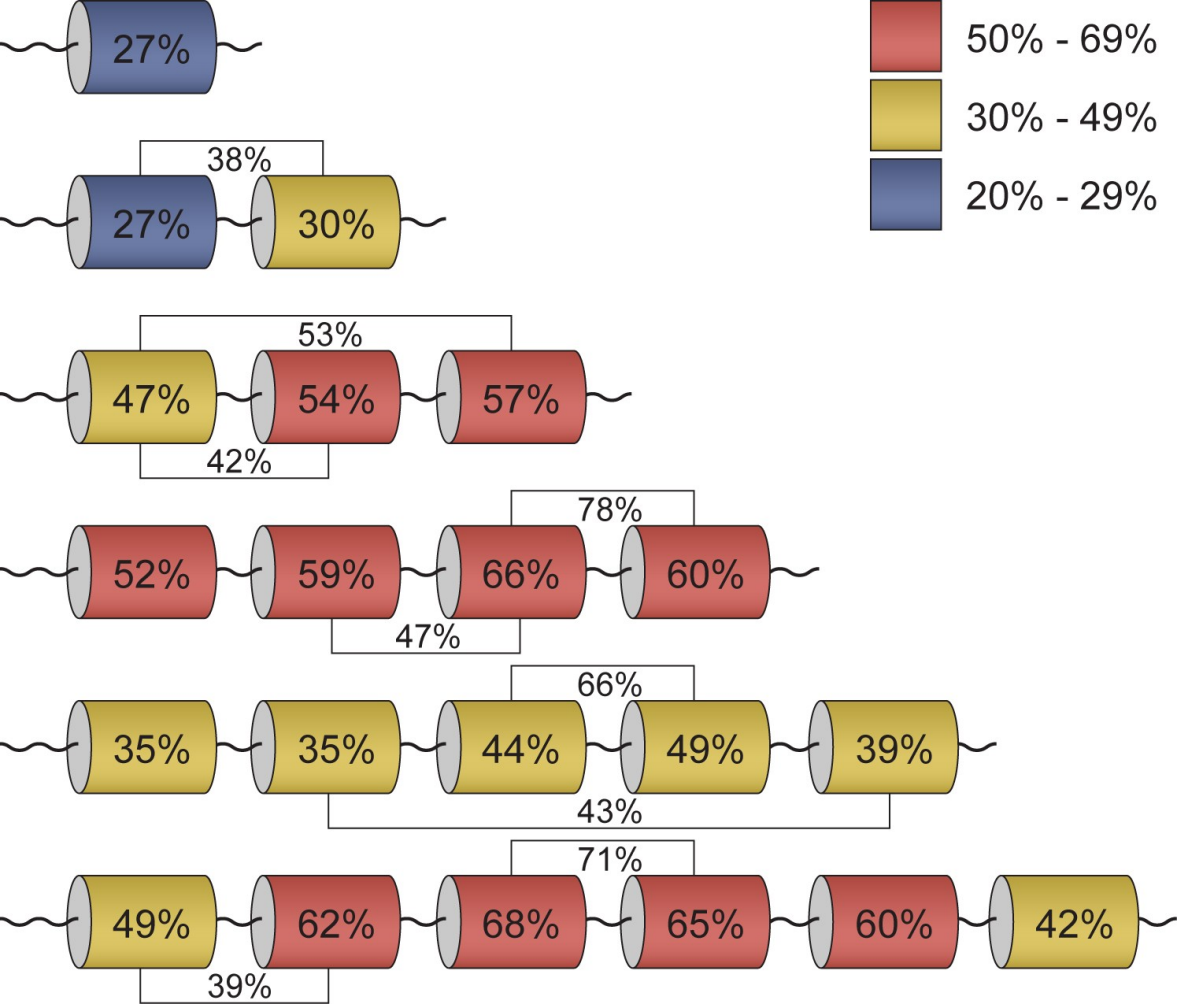
Conservatism of S1 domains within the family of 30S ribosomal protein S1. Average percentage of identity between each domain as well as all domains in proteins containing different numbers of domains is given. Domains with the highest and lowest sequence identity between each other are marked. Percentages of identity for these domains are given in the figure.

To check the equivalence of domain characteristics, each S1 domain in the corresponding S1 protein sequence in different groups (according to the domain number) was aligned in pairs (http://bioinfo.protres.ru/other/pairwise_alignment.xlsx) using the Pairwise2 module from BioPython. The percentages of identity for these domains were calculated using standard parameters. The maximal and minimal values of identity for each group are marked in Fig 5. Domains with the same domain No. in each group were repeatedly aligned (http://bioinfo.protres.ru/other/multiple_alignment.xlsx) using the Clustal Omega service (https://www.ebi.ac.uk/Tools/msa/clustalo/). The obtained results are also shown in Fig 5.

It is noteworthy that S1 proteins containing one-domain have a low percentage of identity among themselves (27%), as well as in one phylum (Tenericutes). Nevertheless, the predicted secondary structures for these sequences [29] are very similar and represent the classic OB-fold. This fact may indicate that for the functioning of proteins related to one-domain S1 proteins, the structural scaffold is more important than the amino acid sequence. This observation has confirmed the statement about the uniqueness of each individual domain in the one-domain S1 proteins [17].

The first and the second domains of S1 proteins containing two structural domains have 38% identity, and pairs with maximum and minimal values of identity are identified for the remaining domains. The first and second domains in S1 proteins containing two structural domains also have a low percentage of identity in the domains: 27% and 30%, respectively.

For S1 proteins containing three structural domains, the maximal value of identity is revealed between the first and third domains (53%) and the minimal value between the first and the second domains (42%). Wherein, the third domain has the maximal percentage of identity (57%) among other domains for this group.

For S1 proteins containing four structural domains, the maximal value of identity is revealed between the third and fourth domains (78%) and the minimal value of identity between the second and third domains. The third domain has also the maximal percentage of homology (66%) among other domains of this group.

The third and fourth domains in the group of S1 proteins containing five structural domains have the maximal percentage of identity (66%), while the second and fifth domains have the lowest percentage of identity (43%). In this group, the fourth domain has the maximal percentage of identity among other domains (49%).

For the most represented S1 proteins containing six structural domains, as well as for S1 proteins with four and five domains, the maximal values of identity are identified between the third and fourth domains (71%) and the minimal values between the first and second (39%). It should be noted that the third and fourth domains (in the groups containing three-, four-, five- and six-domain S1 proteins) also have the maximal values of identity with the S1 domain from PNPase and S1 protein sequences with different single-domain S1 proteins. Moreover, the third domain has the maximal percentage of identity among other domains in this group. Thus, the obtained results showed that for long S1 proteins (five- and six-domain ones) the central part of the proteins (the third domain) is more conserved than the terminal domains.

### Possible evolutionary development of the family of 30S ribosomal proteins S1

The problem of understanding the nature of protein repeats, the corresponding functions for each repeat, and their evolution is still unclear. These repeats evolved from a common ancestor, which necessarily contained a single repeat [30]. Some authors suggested that the common ancestor of the family indeed was a single repeat that formed homo-oligomers for effective functional activity [31]. The homo-oligomeric structure of the ancestor may reflect the intrachain repetitive structure of its modern homologue, with the exception of its multi-chain character. But there are examples of homologous multirepeat assemblies, which are formed both from oligomers with single repeats and from one chain of several repeats [30].

**Fig 6:**
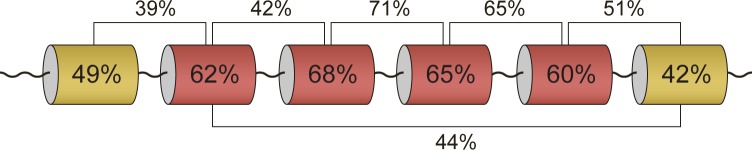
Identity of domains in the six-domain S1 protein. Average percentage of identity between domains and within separate domains is given in the figure.

For the investigated bacterial proteins, the maximum number of S1 domain repeats (six) is sufficient to perform all necessary functions. As shown above, the third domain in this group has the maximal identity (68%) among other domains. In addition, this domain has the highest identity with the S1 domain from PNPase (*E*.*coli*) and S1 domains from one-domain S1 proteins (Tenericutes, Mollicutes) (Fig 6).

As can be seen from Fig 6, the maximal values of identity are identified between the third and fourth domains (71%). Along with the fourth domain, the fifth domain is the most homologous (65%), which in turn has 50% identity with the sixth domain. The identity of the remaining domains (first and second) are below 50%. Thus, the obtained results showed that for six-domain S1 proteins, the central part of the proteins (the third and fourth domains) is more conserved than the terminal domains. In addition, some of the conserved residues Phe19, Phe22, His34, Asp64, and Arg68 [28] are located in the third domain, which once again confirms the uniqueness of this repeat and allows us to consider it as the strongest RNA binding site. Thus, the central part of the proteins (third and fourth domains) is apparently vital for the activity and functionality of these proteins.

This suggestion is consistent with experimental data. One of the well-studied proteins with six S1 domain repeats is the bacterial 30S ribosomal protein S1 from *E*.*coli*. It was shown that cutting one S1 domain from the C-terminus or two S1 domains from the N-terminus of a protein decreases only the effectiveness of protein functions, but not its functional capabilities [21,22].

It should be noted that the bacterial 30S ribosomal protein S1 from parasitic bacteria Mollicutes effectively performs the main functions of RNA-binding [23]. There is an assumption in the literature that mycoplasmas (Mollicutes) are a regressive branch of evolution of some Gram-positive bacteria or clostridia (Firmicutes) [32]. This hypothesis was confirmed experimentally and is considered in two possible variants: all mycoplasmas originate either from a common ancestor with Gram-positive bacteria, or from different bacteria [32]. Based on a comparison of the 16S rRNA oligonucleotide sequences of several species of mycoplasmas and Gram-positive bacteria of the genera *Clostridium*, *Bacillus*, *Lactobacillus*, and *Streptococcus*, a reasonable assumption was made regarding their evolutionary relationship with the division Firmicutes [33,34]. A more detailed analysis of 16S RNA sequences showed that mycoplasmas are phylogenetically closest to clostridia [35]. In turn, the most likely ancestors of clostridia are believed to be Gram-positive bacteria with a low G + C content in their DNA.

In the future, a more detailed analysis of the phylogenetic and evolutionary relationships between one-containing S1 domain proteins and the third and/or fourth domains will allow us to conclude the evolutionary development of the family of bacterial proteins S1 and verify our previous suggestion about the effect of reducing the evolution of the number of repeats in the family of 30S ribosomal proteins S1 [36].

## Discussion

Studies of 1453 S1 sequences available in the UniProt database showed that the number of structural domains in the ribosomal proteins S1 can be considered as a distinctive feature for the phylogenetic grouping of bacteria in 25 different bacterial phyla. It can be assumed that bacteria affiliation may be associated with the structural features and multifunctional activity of ribosomal proteins S1. The obtained results differ from the data obtained for small data sets [17,34], and they should be considered as more accurate. For example, our data show that a large number of bacteria of the phylum Actinobacteria contain four S1 domains, and a smaller number contains five S1 domains. According to [17], all Actinobacteria should be considered as proteins with five domains, with one domain at the C-terminus that is not identified. Also, Proteobacteria contain all possible variants of the number of S1 domains with a predominance of six domain proteins. Such differences are primarily associated with the volume of the samples studied.

Proteins belonging to the phylum Proteobacteria and containing six S1 domains are mainly presented. This fact undoubtedly is associated with the wide distribution of this phylum bacteria and the presence of their sequences in the UniProt database. However, the stability of the number of multiple structural domains in these bacteria is apparently an evolutionary feature, that is necessary for functional diversity. The least represented proteins contain two S1 domains. The sequences found in this group belong to bacteria of phyla Actinobacteria, Firmicutes, and Proteobacteria and are mainly represented by an individual representative in each bacterial class within the phylum.

Verification of the equivalence of domain characteristics showed that for long S1 proteins (five- and six-domain S1 proteins), the central part of the proteins is more conserved than the terminal domains, and, apparently, is vital for the activity and functionality of S1. Moreover, when aligning sequences between individual domains in each group, a rather low percentage of identity is revealed, which indicate that for the general functioning of these proteins the structure scaffold (OB-fold) is obviously more important than the amino acid sequence.

Based on the obtained data, further investigations of possible evolutionary, functional, and structural relationships between bacterial phyla and bacterial classes within each phylum will reveal the relationship between the number of structural repeats and the specificity of the multifunctional activity of proteins of this family. Besides, the study of evolutionary relationships for the considered phyla will allow us to find evidence for one of the proposed theories of the evolutionary development of proteins with structural repeats: from multiple assemblies to single or vice versa.

## Materials and methods

### Construction of ribosomal proteins S1 dataset

To make a representative dataset of records for the family of ribosomal proteins S1 from the UniProt database, all records for the bacteria containing any one of the keywords *«30s ribosomal protein s1»*, *«ribosomal protein s1»*, *«30s ribosomal protein s1 (ec 1*.*17*.*1*.*2)»*, *«30s ribosomal protein s1 (ribosomal protein s1)»*, *«ribosomal protein s1 domain protein»*, *«rna binding protein s1»*, *«rna binding s1 domain protein»*, *«s1 rna binding domain protein»* in the protein name were selected (UniProt release 2018_04). Then the obtained array of data was used to choose only proteins encoded by the rpsA gene or its analog, for example, rpsA_1, rpsA_2, rpsA_3 etc. Only this gene, coding the ribosomal protein S1, in the European nucleotide archive (ENA, http://www.ebi.ac.uk/ena) is affiliated to the STD class, i.e. the class of standard annotated sequences. Therefore, the selection of records for the rpsA gene made it possible to regard the obtained collection as reliable, complete and sufficient for the aim of the study. From the obtained dataset records with six-digital identification numbers (annotated records in the UniProt database) were selected. All data were collected in one file that was the basis for further analysis, namely for collection of data on the number of structural domains and for phylogenetic grouping in the main bacterial phyla (http://bioinfo.protres.ru/other/uniprot_ids.xlsx). Records characterized by the presence of the word “candidate” were removed from our dataset, because there is not enough information for such records to call it a new species and define phylum according to the International Code of Nomenclature of Bacteria.

### Number and identification of structural domains in protein sequences

Four databases of protein domains, SMART (about 1200 domains), Pfam (16295 families of proteins united in 559 clans), PROSITE (1775 models, 1174 profiles and 1195 rules), and SUPFAM (1962 protein domains and 3245 different types of organisms) were analyzed in the study. The values of the number of domains S1 corresponding to the data from each database were selected for each analyzed record. If no data on the number of domains in one of the analyzed bases were available (None), this number was taken to be zero. Profiles of additional domains (not S1) according to their sequences were taken from the database InterPro (http://www.ebi.ac.uk/interpro/).

### Number and identification of structural domains in protein sequences

The values of the number of S1 domains corresponding to the SMART database (about 1200 domains), were selected for each analyzed record. If no data on the number of domains in one of the analyzed bases were available (None), this number was taken to be zero (these records were removed from investigated dataset). Accurate borders for each S1 domain for each record were taken from the UniProt database (position, domain and repeats field). Records with additional domains (not S1) according to the InterPro database (http://www.ebi.ac.uk/interpro) were also removed from the investigated dataset.

### Taxonomic diversity of bacteria

Bacteria were classified in main taxonomic categories (phylum, class, family, genus, type) in accord with the Taxonomic database NCBI (http://www.ncbi.nlm.nih.gov/taxonomy)

### Alignment

A global pairwise sequence alignment (Needleman-Wunsch algorithm) using a dynamic programming algorithm was used. The Multiple Sequence Alignment was implemented by the Clustal Omega service (https://www.ebi.ac.uk/Tools/msa/clustalo/). Clustal Omega is a multiple sequence alignment program that uses seeded guide trees and HMM profile-profile techniques to generate alignments between three or more sequences. In our work standard parameters of this program were used.

### Realization

Algorithms of search, collection, alignment, representation and analysis of the data were realized using the freely available programming language Python 3 (https://www.python.org/). Pairwise2 module from Biopython was used for the alignment functions to get global alignments between two sequences. Bio.pairwise2 uses the Smith-Waterman algorithm for local alignment, and Needleman-Wunsch for global alignment with standard parameters.

## Supporting information

S1 Table Comparison of data on representation (the number of different records) for the analyzed phyla of bacteria in the family of ribosomal proteins S1 for four databases of protein domains. (DOCX)
