# Supplementary material for: The number of domains in the ribosomal protein S1 as a hallmark of the phylogenetic grouping of bacteria
Source: PLoS One. 2019 Aug 22;14(8):e0221370. doi: 10.1371/journal.pone.0221370 (PMC6705787; doi:10.1371/journal.pone.0221370)
Supplement: S1 Table — (DOCX) [file pone.0221370.s001.docx]

**Table 1.** **Comparison of data on representation (the number of different records) for the analyzed phyla of bacteria in the family of ribosomal proteins S1 for four databases of protein domains**

| № | Phylum | SMART | SUPFAM | Pfam | PROSITE |
| --- | --- | --- | --- | --- | --- |
| 1 | Acidobacteria | 1 | 1 | 1 | 1 |
| 2 | Actinobacteria | 746 | 746 | 746 | 746 |
| 3 | Aquificae | 1 | 1 | 1 | 1 |
| 4 | Bacteroidetes | 106 | 107 | 107 | 107 |
| 5 | Caldiserica | 1 | 1 | 1 | 1 |
| 6 | Chlamydiae | 58 | 58 | 58 | 58 |
| 7 | Chlorobi | 1 | 1 | 1 | 1 |
| 8 | Chloroflexi | 2 | 2 | 2 | 2 |
| 9 | Cyanobacteria | 28 | 28 | 28 | 28 |
| 10 | Deferribacteres | 1 | 1 | 1 | 1 |
| 11 | Deinococcus-Thermus | 6 | 6 | 6 | 6 |
| 12 | Elusimicrobia | 1 | 1 | 1 | 1 |
| 13 | Fibrobacteres | 1 | 1 | 1 | 1 |
| 14 | Firmicutes | 517 | 521 | 520 | 519 |
| 15 | Fusobacteria | 1 | 1 | 1 | 1 |
| 16 | Gemmatimonadetes | 1 | 1 | 1 | 1 |
| 17 | Haloplasmatales | 1 | 1 | 1 | 1 |
| 18 | Ignavibacteriae | 1 | 1 | 1 | 1 |
| 19 | Nitrospinae/Tectomicrobia | 1 | 1 | 1 | 1 |
| 20 | Nitrospirae | 1 | 1 | 1 | 1 |
| 21 | Planctomycetes | 5 | 5 | 5 | 5 |
| 22 | Proteobacteria | 2342 | 2345 | 2347 | 2340 |
| 23 | Spirochaetes | 21 | 21 | 21 | 21 |
| 24 | Synergistetes | 1 | 1 | 1 | 1 |
| 25 | Tenericutes | 10 | 16 | 10 | 13 |
| 26 | Thermotogae | 1 | 1 | 1 | 1 |
| 27 | Verrucomicrobia | 1 | 1 | 1 | 1 |
|  | **Total amount of records** | 3857 | 3871 | 3866 | 3861 |
